# Supplementary material for: Factors related to the intention of choosing shared E-scooters for metro transfer: A survey study integrating weather perception into satisfaction evaluation from Changsha
Source: PLoS One. 2024 Sep 9;19(9):e0309953. doi: 10.1371/journal.pone.0309953 (PMC11383246; doi:10.1371/journal.pone.0309953)
Supplement: S2 File — (DOCX) [file pone.0309953.s003.docx]

**Supporting information**

**S1. Table.** Questionnaire survey on shared e-scooters transfer to the metro

Dear participants:

Greetings! Thank you very much for your participation in this survey. The purpose of this questionnaire is to understand citizens' preference for using shared e-scooters to complete the "last-mile" metro transfer in their daily trips. Based on your responses, we will analyze and evaluate the advantages and disadvantages of shared e-scooters, as well as citizens' considerations and needs for choosing a metro transfer method. Your valuable opinions will provide important support to our research, which will help improve public transportation services and meet consumer needs.

Please be assured that this survey is completely anonymous and the data collected will only be used for this study and will not be disclosed to any third party. By filling out this questionnaire, you are deemed to have agreed to participate in the survey. Please fill out the questionnaire truthfully according to your actual situation, and try to be as objective and truthful as possible when answering.

The following questions are single-choice, please put a "√" in corresponding position.

1. Gender: □Male □Female
2. Age: □Below 18 □18-25 □26-35 □36-50 □51-60 □Above 60
3. Education attainment: □High school and below □Junior college and vocational high school □undergraduate □Master and doctor
4. Your career:

□Student □Government □corporation □Boss/executive □Freelance work

□retirement □other

1. Monthly salary: □Under 3k □3k-6k □6k-10k □10k-20k □Over 20k
2. How far do you ride shared e-scooter on average?

□<1km □1-2km □2-4km □>4km

1. How long do you ride hared e-scooter on average?

□Within 5min □5-15min □15-30min □30-45min □More than 45min

1. Average time spent on the metro:

□Within 15min □15-30min □30-45min □More than 45min

1. Frequency of riding shared e-scooters per week:

□Occasionally □1-2 times a week □3-5 times a week □6-10 times a week

□More than 10 times a week

Here are some of your feelings about shared e-scooters, please put a "√" in corresponding position according to your feelings.

1. (COU1) shared e-scooters are neatly arranged.

□Strongly Disagree □Disagree □Neutrality □Agree □Strongly Agree

1. (COU2) Easy to pick up by scanning a code

□Strongly Disagree □Disagree □Neutrality □Agree □Strongly Agree

1. (COU3) Easy payment

□Strongly Disagree □Disagree □Neutrality □Agree □Strongly Agree

1. (COU4) Shared e-scooters make travel easy

□Strongly Disagree □Disagree □Neutrality □Agree □Strongly Agree

1. (COU5) Using an electric car is easy for me

□Strongly Disagree □Disagree □Neutrality □Agree □Strongly Agree

1. (SAF1) Motor vehicles are courteous when mixing with them

□Strongly Disagree □Disagree □Neutrality □Agree □Strongly Agree

1. (SAF2) Well traffic order

□Strongly Disagree □Disagree □Neutrality □Agree □Strongly Agree

1. (SAF3) Little interference in riding

□Strongly Disagree □Disagree □Neutrality □Agree □Strongly Agree

1. (SAF4) Little interference from pedestrians

□Strongly Disagree □Disagree □Neutrality □Agree □Strongly Agree

1. (SAF5) Well-separated between motor vehicles and bikes

□Strongly Disagree □Disagree □Neutrality □Agree □Strongly Agree

1. (PEOU1) Adequate width of non-motorized lanes

□Strongly Disagree □Disagree □Neutrality □Agree □Strongly Agree

1. (PEOU2) Smooth road without bumps

□Strongly Disagree □Disagree □Neutrality □Agree □Strongly Agree

1. (PEOU3) Continuous non-motorized lanes with few interruptions or sudden narrowing

□Strongly Disagree □Disagree □Neutrality □Agree □Strongly Agree

1. (PEOU4) Shade trees along the way

□Strongly Disagree □Disagree □Neutrality □Agree □Strongly Agree

1. (PEOU5) Clear bicycle guidance signs

□Strongly Disagree □Disagree □Neutrality □Agree □Strongly Agree

1. (COM1) Save effort

□Strongly Disagree □Disagree □Neutrality □Agree □Strongly Agree

1. (COM2) Little riding disruption

□Strongly Disagree □Disagree □Neutrality □Agree □Strongly Agree

1. (COM3) Fewer bad e-scooters

□Strongly Disagree □Disagree □Neutrality □Agree □Strongly Agree

1. (COM4) Comfortable seat

□Strongly Disagree □Disagree □Neutrality □Agree □Strongly Agree

1. (SAT1) Riding it’s a healthy and environmental way

□Strongly Disagree □Disagree □Neutrality □Agree □Strongly Agree

1. (SAT2) I am willing to recommend it to friends and family

□Strongly Disagree □Disagree □Neutrality □Agree □Strongly Agree

1. (SAT3) Enhanced living quality

□Strongly Disagree □Disagree □Neutrality □Agree □Strongly Agree

1. (SAT4) Affordable

□Strongly Disagree □Disagree □Neutrality □Agree □Strongly Agree

1. (LOF1) I will still choose it even if the price is properly increased

□Strongly Disagree □Disagree □Neutrality □Agree □Strongly Agree

1. (LOF2) I will still choose it even if the pick-up and return is a little further away

□Strongly Disagree □Disagree □Neutrality □Agree □Strongly Agree

1. (LOF3) I will still choose it even if the bus is convenient

□Strongly Disagree □Disagree □Neutrality □Agree □Strongly Agree

1. (LOF4) I ride a lot

□Strongly Disagree □Disagree □Neutrality □Agree □Strongly Agree

1. (TRA1) Reasonable layout of metro entrances and exits

□Strongly Disagree □Disagree □Neutrality □Agree □Strongly Agree

1. (TRA2) It's more convenient than taking public transportation.

□Strongly Disagree □Disagree □Neutrality □Agree □Strongly Agree

1. (TRA3) Proximity to pick-up and return points

□Strongly Disagree □Disagree □Neutrality □Agree □Strongly Agree

1. (TRA4) Sufficient e-scooters

□Strongly Disagree □Disagree □Neutrality □Agree □Strongly Agree

1. (TRA5) Ample parking spaces

□Strongly Disagree □Disagree □Neutrality □Agree □Strongly Agree

1. (WEA1) Foggy weather does not affect riding

□Strongly Disagree □Disagree □Neutrality □Agree □Strongly Agree

1. (WEA2) Light rain weather does not affect riding

□Strongly Disagree □Disagree □Neutrality □Agree □Strongly Agree

1. (WEA3) Hot weather does not affect riding

□Strongly Disagree □Disagree □Neutrality □Agree □Strongly Agree

1. (WEA4) Cold weather does not affect riding

□Strongly Disagree □Disagree □Neutrality □Agree □Strongly Agree
