# Supplementary material for: Factors related to the intention of choosing shared E-scooters for metro transfer: A survey study integrating weather perception into satisfaction evaluation from Changsha
Source: PLoS One. 2024 Sep 9;19(9):e0309953. doi: 10.1371/journal.pone.0309953 (PMC11383246; doi:10.1371/journal.pone.0309953)
Supplement: Attachment — Submitted filename: Response to Reviewers.docx [file pone.0309953.s004.docx]

Reviewer #1: The manuscript sounds technically and uses questionnaire data for statistical analysis, which can basically support the conclusions. The authors have provided a detailed description of the data processing, and presented it in the form of simple tables. However，some issues should be considered.

1. The writing of the manuscript needs improvement with the assistance of English editing, cause some grammatical, styling, and typos are found in the manuscript.

**Response:** Thank you for your suggestions. We have made improvements to the manuscript and had an English editor revise our article. All changes are marked and do not affect the content or structure of the paper. We sincerely appreciate the efforts of the Editors and Reviewers and hope the revisions meet with your approval.

2. The format is inconsistent when arranging the introduction, eg. the interrogative sentence in the second paragraph of (1) should be placed at the end of (1) to enhance the readability of the manuscript.

**Response:** Thank you for your suggestion. We have addressed the issue and reviewed the rest of the paper.

3. The reference format needs to be optimized (e.g. reference [46] is incomplete), and the clarity of the images needs to be improved through the manuscript.

**Response:** Thank you for your suggestion. We have corrected the references and improved the clarity of the figures. The full text has also been rechecked to ensure that similar problems do not recur.

4. It is necessary to point out the link source when citing data.

**Response:** Thank you for your suggestion. We have rechecked the full text and added link source in all locations where reference data is cited.

5. The suggestion to set up a changing room should be explained more detailed, which needs to be specifically considered.

**Response:** Thank you for your suggestions. We have provided a more detailed explanation of the proposed changes from two aspects.

(1) We have included additional literature to support the proposal for change rooms, citing three relevant studies which are " Tuli F M, Mitra S, Crews M B. Factors influencing the usage of shared E-scooters in Chicago[J]. Transportation research part A: policy and practice, 2021, 154: 164-185.", " Burke M I. Are cycle centers effective transport interventions? Evaluating king george square cycle center in Brisbane, Australia[J]. Transportation research record, 2011, 2247(1): 118-125.", " Tin Tin S, Woodward A, Thornley S, et al. Cyclists’ attitudes toward policies encouraging bicycle travel: findings from the Taupo Bicycle Study in New Zealand[J]. Health Promotion International, 2010, 25(1): 54-62.".

(2) We have added an analysis and explanation of the reasonableness of the proposal in Section 6.2.1. First, we argue that the Shared E-scooter (SE) serves as a means of transportation for open-air travel. However, extreme weather conditions such as excessively cold/hot or rainy/snowy weather can deter users from choosing SE as their mode of travel due to the discomfort it may cause. Additionally, users may have the need to freshen up their appearance or adjust their clothing after riding. Installing a change room in the office can increase the likelihood of using SE for travel under such circumstances. Second, according to the survey data presented in this paper, a larger proportion of SE users are commuters, who typically exhibit a more stable and loyal pattern in selecting their mode of transportation. However, the necessity of wearing formal attire at work can pose a significant discomfort for users riding SE in formal clothes, reducing their loyalty and likelihood of choosing SE. By incorporating changing rooms in the workplace, the issue of changing clothes can be effectively addressed, thus mitigating the obstacles to riding to a certain extent. This improvement measure is therefore highly feasible.

6. At the beginning of the manuscript, gender, age, occupation, and other characteristics are distinguished in the sample features, but the differences between different feature samples are not considered in the subsequent analysis.

**Response:** Thank you for your suggestion. It is indeed necessary to discuss the heterogeneous choice preferences of different groups based on personal attributes, and the absence of this section was an oversight on our part. We have supplemented the analysis of the psychological mechanisms influencing SE transfer in Section 6.1, focusing on gender, education level, age, and occupation. Based on these analyses, we propose improvement priorities for the Shared E-scooter System (SES) tailored to the characteristics of different groups. These priorities are summarized in the following conclusions.

(1) The female group cares about the impact of riding on their image, has a lower tolerance threshold for weather, and is also more critical of riding safety and road conditions. In the future, loyalty to SE can be enhanced by leveling and ensuring continuity in non-motorized lanes, as well as providing raincoats, among other measures. However, men show a stronger overall interest in this emerging mode of travel, and improvements in all areas related to SE could strengthen their propensity to choose this transfer mode.

(2) Aiming at the decision-making psychology of different education groups, the M4 model established in this paper shows excellent fitting effects in both groups. Among them, the model's explanatory power is more prominent in the undergraduate and above group, indicating that the highly educated population is more likely to accept emerging travel modes. Measures to simplify the operation of the system and increase the number of SE can further enhance loyalty. On the other hand, individuals with a high school or college education are more concerned about individual feelings such as riding safety, comfort level, and weather conditions. They make their choices after considering these factors.

(3) The respondents were categorized into two groups, Generation Z and Millennials, based on the age of users. Young people who are new to society use SE to transfer to the metro, and therefore attach more importance to the infrastructure and service level of SE. The use scenario for middle-aged users is daily commuting, characterized by stable and continuous travel, so changes in the external environment will not significantly interfere with their travel choices. It should be noted that overly complex steps greatly hinder the use of SE by older people, so simplifying the operation process will promote the adoption of this transfer mode.

(4) Analyzing the user's occupation, college students, bosses/executives, and other freelance groups have more discretionary time, leading them to pursue high-quality travel experiences. They make their selection after comprehensively evaluating all aspects of the conditions. On the other hand, non-freelance groups such as corporate employees have stable travel patterns, but their fatigue prompts them to pay more attention to the riding experience, especially riding comfort, safety, and other perceptual indicators. Subsequent optimizations can be made to improve riding configuration and traffic order.

7. how to test the applicability and effectiveness of the M4 model among different samples should be detailed.

**Response:** Thank you for your suggestion. We apologize that we did not differentiate between the test set and validation set data during the questionnaire collection process, which is a missing element in our previous study. To address this, we randomly divided the survey data from the 683 respondents into a test set (containing 456 samples) and a validation set (containing 227 samples) at a ratio of 2:1. We then applied the M4 model to each set for calculation. The results in Table 1 indicate that the difference in fitting effect between the two sets is not significant, suggesting that the model exhibits good stability. While this validation method has inherent limitations, it can still to some extent demonstrate the availability and validity of the model across different samples.

Table 1 Comparison of models in test set and validation set

| Data  Indicators | Test  set | Validation  set | Percentage  difference | Data  Indicators | Test  set | Validation  set | Percentage  difference |
| --- | --- | --- | --- | --- | --- | --- | --- |
| GoF | 0.347 | 0.317 | 9.46% | Total effect of path | - | - | - |
| SRMR | 0.153 | 0.156 | -1.92% | PEOU -> LOF | 0.105 | 0.093 | 12.90% |
| f2 | - | - | - | PEOU -> SAT | 0.312 | 0.354 | -11.86% |
| PEOU -> SAT | 0.094 | 0.083 | 13.25% | PU -> COM | 0.723 | 0.697 | 3.73% |
| PU -> COM | 1.097 | 1.043 | 5.18% | PU -> COU | 0.593 | 0.548 | 8.21% |
| PU -> COU | 0.437 | 0.429 | 1.86% | PU -> LOF | 0.296 | 0.333 | -11.11% |
| PU -> LOF | 0.064 | 0.058 | 10.34% | PU -> SAF | 0.712 | 0.748 | -4.81% |
| PU -> SAF | 1.026 | 1.072 | -4.29% | PU -> SAT | 0.268 | 0.312 | -14.10% |
| PU -> SAT | 0.07 | 0.065 | 7.69% | PU -> TRA | 0.684 | 0.606 | 12.87% |
| PU -> TRA | 0.863 | 0.779 | 10.78% | SAT -> LOF | 0.338 | 0.334 | 1.20% |
| SAT -> LOF | 0.128 | 0.143 | -10.49% | WEA ->LOF | 0.212 | 0.238 | -10.92% |
| WEA -> LOF | 0.048 | 0.054 | -11.11% | WEA ->PEOU | 0.184 | 0.217 | -15.21% |
| WEA -> PEOU | 0.035 | 0.044 | -20.45% | WEA ->SAT | 0.057 | 0.049 | 16.33% |

Reviewer #2:

1.The quality of the current images needs improvement. High-quality images can better showcase the content.

**Response:** Thank you for your suggestion. We have exported all full-text images as vector graphics to improve image clarity.

2.The table analysis in the article's conclusion is not very intuitive and could be presented more clearly using more figures and graphics.

**Response:** We sincerely apologize for this issue. To enhance the readability of the article, we have converted Table 3 from the initial version into a percentage stacked bar chart (i.e., Figure 2 in the newly submitted version, which can be seen in Figure 1 below,). This visualization illustrates the distribution of SE users' social attributes through a proportional relationship. Additionally, we have changed the format of Table 6 from the initial version to a bar chart (i.e., Figure 3 in the newly submitted version, which can be seen in Figure 2 below), which presents the dimension scores and stability using values.

Figure 1 Descriptive of sample characteristics

Figure 2 Scores for each dimension

3.The utilized data only spans two days. Is this sufficient to this study?

**Response:** Thank you for your suggestions. Although the questionnaire survey in this paper only lasted for two days, we conducted a pre-survey before the formal questionnaire survey. The final 20 survey locations were selected to cover various types of travel environments, such as residences, businesses, offices, schools, transportation hubs, parks, etc., making the sample data collection scenarios more comprehensive. Moreover, this study does not involve time series analysis, and the amount of survey data is sufficient for our needs. However, your comments have inspired us, and we plan to continue conducting annual surveys in subsequent studies to analyze the trends in choices of SE users over longer time spans. This will lead to research outcomes that have a time-series character.

4. The article mentions that the sample includes a high proportion of young people and participants with higher levels of education, which may affect the representativeness of the results. It is recommended that the authors discuss the potential impact of sample bias on the research findings.

**Response:** We sincerely apologize for this problem and have made corresponding changes in the relevant sections.

(1) In Section 4.2, we added relevant explanatory text to the data statistical analysis. It is highlighted that the results of the questionnaire survey (e.g., gender ratio, age distribution, education level, etc.) exhibit consistent user composition characteristics with the SE customer group report "Songguo Mobility. 2023 Shared Electric Vehicle Riding Report[R], 2023". Therefore, the proportional differences presented in the sample data only reflect the natural selection preference for SE of a specific group, rather than subjective survey sample bias. The results of the study represent the current state of market composition and user group characteristics.

(2) Thank you for your question, it is very enlightening. Although the sample data is consistent with the SE market composition, a small percentage of older and less educated groups were included, which may result in overlooking some needs of this group in the proposed promotional measures in this paper. We have added this aspect of the limitations in Section 6.4 and plan to focus on target groups such as older or less educated individuals and tourists in subsequent studies to promote SE travel comprehensively.

5.The current method is the Technology Acceptance Model (TAM). Maybe other analytical methods can be added for comparison to reflect the universality of the analysis?

**Response:** Thank you for your suggestions. To demonstrate the generalizability of the analysis results, we initially added the TPB model as an alternative research methodology in Section 4.1. TPB is a common methodology for measuring specific subjective behaviors, widely used in travel choice research. Next, we specifically discussed the applicability value of the research methodology in Section 4.3.3. The TPB model was found to be unable to meet the requirements for discriminant validity, indicating that the method could not be adapted to the joint travel scenario of SE transfer to the metro. It is further demonstrated that the TAM model is the more appropriate analytical approach for SE transfer studies.

6.Could the authors discuss potential limitations of applying the TAM model to the context of Shared E-scooter (SE) usage?

**Response:** Thank you for your suggestion. SE has the complex characteristics of combining "emerging" and "traditional". On the one hand, SES, as an emerging mode of transportation developed in recent years, still has a relatively low usage coverage. The system operation mode, usage characteristics, supporting facilities, and technical details all have the characteristics of "new technology and new product". However, on the other hand, from the perspective of transportation, SE is the e-scooter which has been widely used. The traditional mode of transportation is more reflected in the riding method and traffic flow impacts. As you mentioned, from this perspective, there are limitations to the analysis using the TAM model, and it seems more appropriate to conduct the study using the TPB model. As you queried in the fifth question, we included the TPB method in our study and the analysis results proved that TAM is relatively superior in general. However, your suggestion is of great reference value, and we plan to build a hybrid model to carry out the study (e.g., the UTAUT model) in the follow-up study to make up for the limitations that exist in the TAM model.

7.There are also some minor issues:
-Improve the overall layout quality of the document, such as ensuring that Tables 2-6 do not span across pages.
-Table 9 is too wide.

**Response:** We would like to apologize for this problem and have made the following changes to the tables throughout the text.

(1) With the criterion of presenting the main content completely and not affecting the understanding of the article, we streamlined the presentation of the model calculation process. Secondly, for Tables 8 and 9 in the initial version, which show the results of model relevance, reliability and validity, we deleted the data related to the Composite Reliability (CR) and AVE metrics.

(2) Table 3 from the initial version has been transformed into a percentage stacked bar chart (i.e., Figure 2 of the new submitted version, which can be seen in Figure 1 above) to display the distribution of the sample data more intuitively. Table 6, representing the scores for each dimension, has been split into two parts. The main body has been modified into a bar chart (i.e., Figure 3 of the new submitted version, which can be seen in Figure 2 above). The questionnaire items and specific values not reflected in the main body will be uploaded into the "Supporting Materials."

(3) As mentioned in response to question five, we analyzed the applicability of the research methodology in Section 4.3.3, with the addition of discriminant validity results of the TPB model in Table 4 in the latest submitted version.
